# Personal and behavioral correlates of total and domain-specific sedentary behaviors in older Taiwanese adults

**DOI:** 10.1186/s12877-018-0987-9

**Published:** 2018-11-29

**Authors:** Shao-Hsi Chang, Ming-Chun Hsueh, Yung Liao

**Affiliations:** 1 Department of Physical Education, National Taiwan Normal University, 162, Heping East Road Section 1, Taipei, Taiwan; 2 Department of Health Promotion and Health Education, National Taiwan Normal University, Taipei, Taiwan

**Keywords:** Computer use, Transportation, Leisure time physical activity, Sitting, Sociodemographic, Taiwan, Older adults

## Abstract

**Background:**

Evidence of the harmful health effects of sedentary behavior is emerging; however, little is known about domain-specific sedentary behavior correlates. Thus, in this study, the personal and behavioral correlates of total and domain-specific sedentary behavior in older Taiwanese adults were identified.

**Method:**

The sample comprised 1046 older adults (aged ≥65 years). Cross-sectional data on self-administered personal behavioral variables and time spent engaging in domain-specific sedentary behavior were obtained using computer-assisted telephone-based interviews. Binary logistic regression analyses were performed.

**Results:**

Those aged older than 75 years were less likely to have longer total sedentary, computer use, and transportation times. Compared to women, older men were more likely to have longer total sedentary and transportation times. Older adults with low educational levels were less likely to have longer total sedentary and computer use times but were more likely to have an excessive television (TV) viewing time (≥2 h/day). Older adults who lived alone and were overweight had a longer TV viewing time. Furthermore, unemployment was associated with an excessive TV viewing time and shorter transportation time. Older adults residing in nonmetropolitan areas had lower total sedentary, TV viewing, and computer use times. Older adults who engaged in insufficient leisure time physical activity were more likely to have longer total sedentary and transportation times.

**Conclusions:**

Both common and distinct personal and behavioral factors were associated with total and domain-specific sedentary behavior. Interventions for reducing total and domain-specific sedentary behavior should focus on both common and distinct subgroups of the Taiwanese older population.

## Background

Because the proportion of older adults is growing worldwide, health policymakers face substantial challenges in promoting health among older adults [1]. The health benefits of physical activity in older adults has been documented broadly and consistently [2]. Recently, sedentary behaviors (i.e., too much sitting, which is distinct from too little exercise) were revealed to be crucial for the cardio-metabolic health of older adults [3]. Furthermore, research has indicated that older adults who regularly sit for prolonged durations have a higher risk of obesity, metabolic syndrome, and all-cause mortality, independent of moderate-to-vigorous and leisure time physical activity (LTPA) [4, 5]. Sedentary behavior, a new research topic in public health, is distinct from insufficient physical activity, and involves prolonged periods of sitting during activities that entail low levels of energy expenditure (1.0–1.5 metabolic equivalents, METs) such as television (TV) viewing, computer use, and the use of motorized transport [6]. Notably, previous studies have evidenced that older populations (≥65 years) engage in higher levels of sedentary time than younger populations [7]. Therefore, tailored and effective strategies to reduce sedentary behavior are required for disease prevention and health promotion among older adults.

According to a behavioral epidemiology framework, an in-depth understanding of the personal factors associated with sedentary behavior is critical for designing relevant policies and tailored interventions for at-risk populations [6]. A recent systematic review summarized evidence from numerous countries regarding the determinants of sedentary behavior in older adults, and indicated that personal characteristics (e.g., age, gender, educational attainment, and living status) were associated with sedentary behavior [8]. However, previous studies have mainly focused on TV viewing time or total sedentary time [9–11]. Although the significance of domain-specific sedentary behavior was previously emphasized for the development of effective interventions [12], few studies have examined the associations between personal factors and other domain-specific sedentary behaviors (e.g., computer use and use of motorized transport) [13]. Moreover, to the best of our knowledge, only one study on older adults, which was conducted in Belgium, reported the existence of relationships between personal factors and different domain-specific sedentary behaviors (i.e., TV viewing, computer use, and the use of motorized transport) [13]. At the time of writing, no study has investigated the personal factors related to different domain-specific sedentary behaviors in older adults in Asia. Therefore, studies on sedentary behavior among older adults living in different countries are warranted, because the relationship between personal attributes and sedentary behavior might vary according to culture and environment [7].

Older adults might have more time to allocate to physically engaging leisure activities, and hence have many opportunities to achieve and maintain the recommended levels of physical activity [14]. Moreover, LTPA is a critical behavioral factor in improving health in older adults [1]. Although LTPA was previously emphasized as playing a role in replacing sedentary behavior, and thus leading to superior physical functioning [15], the associations of LTPA with different sedentary behaviors in older adults remain unclear [8]. In previous studies, several measures of LTPA have been revealed to have significant associations with sedentary behavior [9–11, 15]; however, in one study, no association was observed between objectively-measured physical activity and sedentary behavior in older adults [16]. Therefore, considering the need to identify correlations between LTPA and different domain-specific sedentary behaviors in older adults, the purpose of this study was to determine the associations of personal and behavioral factors with total and domain-specific sedentary behaviors in older Taiwanese adults.

## Methods

### Participants

In May 2015, a cross-sectional survey was conducted by a telephone-based research service company in Taiwan using a computer-assisted telephone interview system. Samples were randomly stratified by gender and municipality (i.e., Taipei City and Chiayi County). Sampling was performed using a random-digit-dialing telephone-based survey. With a sampling error of 3% and a confidence interval (CI) of 95%, 1068 older adults were required to achieve a sufficient sample size and statistical power. A total of 1714 older adults were interviewed, and 1095 of the interviewees completed the survey (response rate: 63.9%). The older adults were provided with a research statement that fully explained the purpose and content of the research, and verbal informed consent was obtained at the start of the telephone interviews. No incentive was offered to the respondents. This study protocol was approved by the Ethics Committee of the National Taiwan Normal University (201504HM004).

### Sedentary behavior variables

The outcome variable was self-reported sedentary time, evaluated using the Measure of Older Adults’ Sedentary Time questionnaire [17]. Respondents were asked seven items in a 1-week recall questionnaire, in which they reported the total time spent performing each named activity in a sitting or reclining position over the prior week, excluding time spent sleeping. The activities were watching TV; using a computer; reading; socializing; traveling in a motor vehicle or on public transport; engaging in hobbies; and other sedentary activities. The item “other sedentary activity time (open-ended question)” was combined with the item regarding hobbies because participants often reported other sedentary activities as hobbies in this study.

### Personal variables

The personal variables included age (“65–74 years” or “≥75 years”), gender (“woman” or “man”), educational level (“college degree or higher” or “up to high school”), living status (“with family” or “alone”), job status (“employed” or “unemployed”), residential area (“metropolitan” or “nonmetropolitan”), and body mass index (BMI) status (“nonoverweight” or “overweight”). BMI, calculated according to self-reported height and weight, was measured as weight (kg) divided by squared height (m^2^). BMI scores were based on self-reported weight and height data and grouped into two categories: “nonoverweight” (< 24 kg/m^2^) and “overweight” (≥24 kg/m^2^) using Taiwan-specific cutoff points for older adults [18].

### Behavioral variables

LTPA was measured using the long version of the Taiwan International Physical Activity Questionnaire [19]. The participants were asked to recall the frequency and average duration of leisure time vigorous-intensity activity, moderate-intensity activity, and walking performed within the 7 days prior to the questionnaire. Questions included: “During the last 7 days, on how many days did you engage in the activities (vigorous/moderate/walking) in your leisure time?” and “How much time did you usually spend on one of those days engaging in such activities?” The total LTPA duration was classified as either sufficient (≥150 min/week) or insufficient (< 150 min/week) according to the physical activity guidelines for health benefits [20].

### Statistical analysis

Data collected from the 1046 older Taiwanese adults who completed the entire survey were included in the analysis. Binary logistic regression analyses were performed to examine the relationships of each personal and behavioral variable with total and domain-specific sedentary behaviors. Both personal and behavioral correlates were entered simultaneously into the logistic regression model. Because the distribution of sedentary behavior was skewed, the total and domain-specific sedentary behavior was dichotomized into two categories on the basis of the median values: total sedentary behavior (time spent watching TV, using the computer, reading, socializing, using motorized transport, and engaging in hobbies) was categorized into durations of either < 4.1 or ≥ 4.1 h/day. Specific domains, such as TV viewing was categorized into durations of either < 2 or ≥ 2 h/day. Time spent using a computer was categorized into durations of either 0 or > 0 h/day, whereas motorized transport time was categorized into durations of either < 0.3 or ≥ 0.3 h/day. According to a review article [5], the associations of reading, socializing, and engaging in hobbies with health outcomes remain unexplored; therefore, these three domain-specific sedentary behaviors were excluded from the analyses. A *P* value of <.05 was considered statistically significant. All statistical analyses were performed using SPSS Version 24.0 for Windows.

## Results

Table 1 lists the personal variables of the study participants. In this study, 38.5% of the participants were older than 75 years, 46.9% were men, 76.7% received only a high school education, 13.1% lived alone, 80.8% were unemployed, 48.9% lived in nonmetropolitan areas, 41.9% were overweight, and 60.6% engaged in insufficient LTPA. Compared to national population data, the participants in this study had a similar proportion in terms of age (≥75 years: 38.5% vs. 44.0%) and gender (men: 46.9% vs. 46.4%). Other personal variables such as education level (college degree or more: 23.3% vs. 11.3%), residential area (nonmetropolitan: 48.9% vs. 35.0%), and BMI status (overweight: 41.9% vs. 47.9%) were not similar to national population data.

**Table 1:** Participant characteristics

Variable	Category	Sample of This Study		National Data in 2015^a^
		*N* = 1046	%	%
Age	65–74	643	61.5%	56.0%
	≥ 75	403	38.5%	44.0%
Gender	Women	555	53.1%	53.6%
	Men	491	46.9%	46.4%
Education level	College degree or more	244	23.3%	11.3%
	Up to high school	802	76.7%	88.7%
Living status	With family	909	86.9%	---^b^
	Alone	137	13.1%	
Job status	Employed	201	19.2%	---^b^
	Unemployed	845	80.8%	
Residential area	Metropolitan	534	51.1%	65.0%
	Non-metropolitan	512	48.9%	35.0%
BMI (kg/m2)	Non-overweight (< 24)	608	58.1%	52.1%
	Overweight (≥ 24)	438	41.9%	47.9%
LTPA (min/week)	Sufficient (≥ 150)	449	39.4%	---^b^
	Insufficient (< 150)	597	60.6%	
Total sedentary behavior, median (IQR) h/d		4.1 (2.53–6.5)		---^b^
TV viewing, median (IQR) h/d		2.0 (1–3)		---^b^
Computer use, median (IQR) h/d		0.0 (0–0.16)		---^b^
Motorized transport, median (IQR) h/d		0.3 (0–0.57)		---^b^

^a^Data source: [21, 22]; b: National data could not be obtained for comparison with our data

*BMI*: body mass index; *LTPA*: leisure time physical activity; *IQR*: interquartile range. Total sedentary behaviors comprised time spent engaging in TV viewing, computer use, reading, socializing, use of motorized transport, and hobbies

The median total sedentary time was 4.10 (interquartile range [IQR]: 2.53–6.50) hours/day. The median duration of domain-specific behaviors of TV viewing, computer use, and the use of motorized transport were 2.00 (IQR: 1.00–3.00) hours/day, 0 (IQR: 0.00–0.16) hours/day, and 0.30 (IQR: 0.00–0.57) hours/day, respectively.

Table 2 lists the associations of personal and behavioral variables with total and domain-specific sedentary behavior time, which was determined according to an adjusted logistic regression analysis. Participants aged older than 75 years were less likely to have longer total sedentary times (odds ratio [OR] = 0.64; 95% CI: 0.48–0.85), computer usage times (OR = 0.47; 95% CI: 0.33–0.67), and motorized transport usage times (OR = 0.57; 95% CI: 0.43–0.76) compared with the participants aged between 65 and 74 years. Older men were more likely to have longer total sedentary (OR = 1.65; 95% CI: 1.25–2.18) and motorized transport usage times (OR = 1.94; 95% CI: 1.47–2.58) than older women. Older adults with a lower educational level (up to high school) were less likely to have longer total sedentary (OR = 0.47; 95% CI: 0.33–0.67) and computer usage times (OR = 0.27; 95% CI: 0.19–0.40) and were more likely to have longer TV viewing times (OR = 1.72; 95% CI: 1.20–2.47) than were those who received a college education or higher. Older adults who lived alone were more likely to have longer TV viewing times (OR = 1.67; 95% CI: 1.06–2.62) than those who lived with family. Older unemployed adults were more likely to have longer TV viewing times (OR = 1.75; 95% CI: 1.25–2.44) and were less likely to have higher motorized transport usage times (OR = 0.46; 95% CI: 0.32–0.66) than were those who were employed. Nonmetropolitan residents were less likely to have longer total sedentary times (OR = 0.40; 95% CI: 0.30–0.53), TV viewing times (OR = 0.70; 95% CI: 0.52–0.94), and computer use times (OR = 0.38; 95% CI: 0.26–0.54) than metropolitan area residents. Overweight older adults were more likely to have longer TV viewing times (OR = 1.32; 95% CI: 1.02–1.71) than nonoverweight older adults. Older adults with insufficient LTPA were more likely to have longer total sedentary (OR = 1.52; 95% CI: 1.16–1.99) and motorized transport times (OR = 1.35; 95% CI: 1.02–1.78) than were those with sufficient LTPA.

**Table 2:** Logistic regression analyses of personal and behavioral correlates of total and three domain-specific sedentary behaviors

Variable	Total sitting time OR (95% CI)	TV viewing OR (95% CI)	Computer use OR (95% CI)	Motorized transport OR (95% CI)
Age				
Over 75 years (ref: 65–74 years)	0.64 (0.48–0.85)^*^	0.84 (0.64–1.12)	0.47 (0.33–0.67)^*^	0.57 (0.43–0.76)^*^
Gender				
Men (ref: Women)	1.65 (1.25–2.18)^*^	0.96 (0.73–1.26)	1.29 (0.92–1.81)	1.94 (1.47–2.58)^*^
Education level				
Up to high school (ref: College or more)	0.47 (0.33–0.67)^*^	1.72 (1.20–2.47)^*^	0.27 (0.19–0.40)^*^	1.07 (0.73–1.56)
Living status				
Alone (ref: With family)	1.01 (0.65–1.57)	1.67 (1.06–2.62)^*^	0.85 (0.49–1.50)	1.10 (0.69–1.74)
Job status				
Unemployed (ref: Employed)	1.38 (0.97–1.96)	1.75 (1.25–2.44)^*^	1.02 (0.66–1.59)	0.46 (0.32–0.66)^*^
Residential area				
Non-metropolitan (ref: Metropolitan)	0.40 (0.30–0.53)^*^	0.70 (0.52–0.94)^*^	0.38 (0.26–0.54)^*^	0.74 (0.54–1.01)
BMI^a^				
Overweight (ref: Non-overweight)	1.21 (0.93–1.59)	1.32 (1.02–1.71)^*^	0.96 (0.70–1.32)	0.94 (0.72–1.23)
LTPA^b^				
Insufficient (ref: Sufficient)	1.52 (1.16–1.99)^*^	1.12 (0.85–1.46)	1.26 (0.90–1.71)	1.35 (1.02–1.78)^*^

*BMI*, body mass index; *LTPA*, leisure time physical activity; *OR*, odds ratio, *CI*, confidence interval; ref., reference group. ^a^nonoverweight: BMI < 24 kg/m^2^, overweight: BMI ≥ 24 kg/m^2^; ^b^Insufficient LTPA: < 150 min/week, sufficient LTPA: ≥150 min/week; ^*^statistical significance was set at *P* < .05

## Discussion

This is one of the few studies in Asian countries examining the associations of personal and behavioral variables with total and domain-specific sedentary behaviors in older adults. The results of this study support the results of previous studies of regarding the risks for of excessive total sedentary and TV viewing time in older adults [9–11]. Furthermore, this study adds to the literature on older populations at risk of poor health outcomes related to excessive computer and motorized transport usage times. The main finding of this study is that both common and distinct personal and behavioral factors were related to total and domain-specific sedentary behaviors (i.e., TV viewing, computer use, and use of motorized transport). These findings may have critical implications for policymakers and intervention designers by revealing that both common and distinct at-risk populations should be targeted when designing effective interventions to reduce total and domain-specific sedentary behaviors.

The findings of this study regarding the association between age and total sitting time are inconsistent with those of previous studies [11, 13, 14]. The present study demonstrated that adults aged 75 years or older had shorter total sitting times than did those aged 65–74 years. A possible reason for this inconsistency could be that, in the present study, the time spent on total sedentary behavior was measured using a domain-specific sedentary behavior questionnaire [17], which may have been less clear for older adults who may have difficulty remembering time spent engaging in each sedentary behavior in daily life [23]. In addition, previous studies have indicated that computer and motorized transport usage times were higher for relatively younger older adults than for relatively older adults [13, 24, 25]. Therefore, for a more effective evaluation, future studies should measure domain-specific sedentary behavior rather than total sedentary behavior. For the development of intervention methods, these results suggest that those targeting reductions in sedentary behavior should devote special attention to older adults aged 65–74 years, with a key focus on computer and internet usage and motorized transport usage times.

Regarding educational attainment, the results revealed that older adults with a lower educational level were more likely to have excessive TV viewing times and shorter computer use times than those educated to degree level or above. This was consistent with the results of previous studies on Japanese and Belgian older adults [9, 13]. One explanation is that lower education may be associated with limited knowledge (e.g., knowledge of computers and technology) and low income; thus, older adults with lower educational qualifications may have fewer leisure time options and a longer TV viewing time [9]. This suggests that for older adults with a lower education level, lower computer use times might have been compensated by higher levels of TV viewing [13]. However, compared with older adults with higher education levels, older adults with lower educational levels were associated with a shorter total sedentary time in the present study. Older adults with a lower educational attainment might be at an increased risk of certain adverse health outcomes due to their higher levels of TV viewing time. Previous studies have linked higher levels of TV viewing to higher metabolic risks and being overweight [3, 4], whereas higher levels of computer use had a protective relationship with mental health and muscle strength [5, 26]. Based on these findings and our results, to reduce excessive TV viewing time, older adults with low educational levels should be prioritized for targeting in interventions. In addition, the findings of the present study indicate that lower educational levels are associated with shorter total sedentary time. This finding is inconsistent with the study conducted by van Cauwenberg et al. [13], which indicated no association between educational level and objective total sedentary time. Therefore, this finding cannot be completely explained, because evidence regarding the associations between educational levels and total sedentary time in older adult populations is limited. Intervention strategies to reduce total sitting time among older adults with higher educational level remain difficult to identify. Future studies should further examine the sedentary behavior of older adults with higher educational levels.

Regarding job and living statuses, the current results indicated that unemployed older adults had longer TV viewing times and a shorter motorized transport usage times. Additionally, we also revealed that that the older adults who lived alone had longer TV viewing times. These results were similar to those previous studies [8, 9]. Taken together, these results indicated that older adults who are unemployed and live alone may be more likely to have limited social interactions (e.g., participation in volunteer activities), which may have caused prolonged sitting at home, resulting in a shorter motorized transport usage time; however, watching TV may have compensated for low levels of social interaction [9, 10]. This further supports the idea that TV viewing may act as a replacement for intimate social interactions [27]. Therefore, social environment concerns (i.e. social environment, community networks, and social support) should be considered in the development of intervention techniques for the prevention of excessive TV viewing for older adults who were unemployed and live alone. However, future studies should also examine the social environmental factors related to different domain-specific sedentary behaviors in older adults.

Regarding area of residence, older adults living in nonmetropolitan areas exhibited less total sedentary behavior as well as less TV viewing and computer use than those living in metropolitan areas. The present results support those of previous studies in Western countries [10, 24, 28]. This may also explain the finding that nonmetropolitan older adults were more likely to spend less time performing indoor activities [29]. For example, many older Taiwanese adults who live in rural areas are self-employed as farmers or aquaculture workers; these occupations might encourage older adults who live in rural areas to perform more work-related activities in their daily life than urban older adults. Therefore, for older adults living in metropolitan areas who engage in excessive TV viewing, interventions could focus on the substitution of light activities for sitting, which has been associated with superior physical health.

Regarding BMI, older overweight adults had a higher likelihood of watching TV for 2 h/day than did the nonoverweight older adults. This was consistent with the results of previous studies [4, 9]. In addition, no considerable association was observed between BMI and total sitting time during computer and motorized transport usage. These results contribute to the literature concerning the contexts and subdomains of sedentary behaviors [8]. Interestingly, the relationship between BMI and other sedentary behaviors (i.e., computer use and use of motorized transport) appears to be weak. However, being overweight is a risk factor for type 2 diabetes, cardiovascular disease, metabolic syndrome, cancer, and all-cause mortality [30]. These results also have implications for policy makers or intervention designers to develop effective strategies to minimize TV viewing for older overweight adults. Reverse causality should be considered in the context of the present study, because the association between TV viewing and overweight may be bidirectional (i.e. excessive TV viewing my cause higher BMI, and higher BMI may cause more TV viewing).

Regarding LTPA status, older adults with insufficient LTPA times had higher total sedentary times. Consistent with previous Japanese and Canadian studies, lower physical activity was correlated with prolonged sitting time in older adults [9.11]. Finally, the results evidenced that older adults who lacked engagement in LTPA had a higher likelihood of spending more time using motorized transport. This result was similar to that of a previous study, which determined that adults with insufficient activity levels (< 150 min/week) may spend more time driving a car [31]. Therefore, promoting LTPA and physically demanding transport may be acceptable and appropriate approaches to replacing motorized transport and maintaining active living in older adults. Future studies should devise a favorable strategy for encouraging older adults to use physically demanding transport, such as walking or cycling, to reduce motorized transport time.

Several limitations should be considered when interpreting the current findings. First, because the distribution of dependent variables was skewed, this dichotomization inevitably resulted in a loss of information, and the median split implies that the findings are specific to the range of sitting times observed in the current study. Second, the cross-sectional study design limited the conclusions regarding the causality of the observed relationships of personal and behavioral factors with sedentary time. Third, to estimate domain-specific sedentary behavior, this study relied on self-reported measures that are susceptible to errors resulting from different interpretations of the questions [17, 23]. These measurement errors may have caused bias among the associations observed, leading to an underestimation of the true associations. Fourth, the study data did not constitute a nationally representative sample, because the responses were limited to two localities and the study relied on a telephone-based survey. Fifth, other personal variables, such as household income [7, 9] and health status [11], were not measured, which may have affected the results because limited socioeconomic resources and poor health status may result in reduced physical activity and outdoor activity. Sixth, including segments of the population that did not have a household telephone (approximately 7.1% in 2015) was impossible [32]. Moreover, compared with national data, the respondents of this study had higher educational levels, a higher proportion of participants lived in nonmetropolitan areas, and there was a lower prevalence of being overweight. Thus, the results in the present study may not be applicable to the general population.

## Conclusions

Both common and different personal and behavioral factors were associated with total and domain-specific sedentary behavior (TV viewing, computer use, and motorized transport usage). Our findings highlight the potential for tailored interventions to reduce total and domain-specific sedentary time according to the needs of different personal and behavior subgroups of the older adult population. To reduce total sedentary time, older adults aged 65–74 years, male, older adults with higher education levels, older adults in metropolitan areas, and older adults with insufficient LTPA are crucial target groups. To reduce TV viewing time, older adults who had lower education levels, lived alone, were unemployed, resided in metropolitan area, or were overweight could be considered as crucial target groups when developing interventions. To address higher volumes of computer use, particular attention should be given to older adults aged 65–74 years, those with higher education levels, and those who residing in metropolitan areas. When developing interventions targeting reductions in sitting when driving a car or taking public transport, special attention should be devoted to older adults aged 65–74 years, those who are male, those without employment, and those with insufficient LTPA. Our findings provide valuable starting points for determining the most appropriate programs and policies to address total and domain-specific sedentary behaviors as a health risk among older adults. Future studies should examine which approaches are the most acceptable, feasible, and effective for reducing total and domain-specific sedentary time among older adults.

## Abbreviations

BMI: Body mass index
CI: Confidence interval; Ref: referent group
IQR: Interquartile range
LTPA: Leisure time physical activity
OR: Odds ratio
